# Optimization & enhancement of KGeCl_3_-based perovskite solar cells through charge transport layer engineering

**DOI:** 10.1039/d4ra08299k

**Published:** 2025-01-24

**Authors:** Zulqarnain Abbasi, Shayan Tariq Jan, Mamoona Safeer, Muhammad Imran, Anees Ur Rehman

**Affiliations:** a Department of Electrical Engineering, Sarhad University of Information Technology Peshawar 25000 Pakistan; b Department of Electrical Engineering, University of Engineering & Technology Mardan 23200 Pakistan shayantj11@yahoo.com dr.shayan.tariq@uetmardan.edu.pk; c SCME, National University of Science and Technology Islamabad 22220 Pakistan; d Department of Electrical Engineering, Military College of Signals, National University of Sciences and Technology Islamabad Pakistan

## Abstract

The growing demand for efficient, stable, and environmentally friendly photovoltaic technologies has motivated the exploration of nontoxic perovskite materials such as KGeCl_3_. However, the performance of KGeCl_3_-based perovskite solar cells (PSCs) depends heavily on the compatibility of charge transport layers (CTLs) and optimization of device parameters. In this study, six PSC configurations were simulated using SCAPS-1D software, incorporating CTLs such as Alq_3_, CSTO, V_2_O_5_, *n*PB, and Sb_2_S_3_. Key optimization steps included analyzing CTL-perovskite heterojunction compatibility, evaluating band offsets, electric potential distribution, and recombination rates, followed by fine-tuning layer thickness, doping concentration, defect density, electrode work function, and back-end reflectivity. These optimizations significantly reduced recombination losses, enhanced charge carrier extraction, and improved light absorption, leading to substantial performance improvements. The CSTO-KGeCl_3_-*n*PB configuration demonstrated the highest power conversion efficiency (PCE) of 29.30%, outperforming other optimized configurations, such as Alq_3_-KGeCl_3_-*n*PB and Alq_3_-KGeCl_3_-Sb_2_S_3_, which achieved PCE values of 25.19% and 24.87%, respectively. This comprehensive optimization study highlights the potential of KGeCl_3_ as a promising absorber material for PSCs. The findings pave the way for developing efficient, stable, and sustainable photovoltaic solutions, contributing to the advancement of clean energy technologies.

## Abstract 2

The effect of novel charge transport layers on the behavior and performance of KGeCl_3_ perovskite solar cells was investigated with CSTO-KGeCl_3_-*n*PB structure outperforming the rest by achieving 29.3% efficiency.

## Graphical abstract

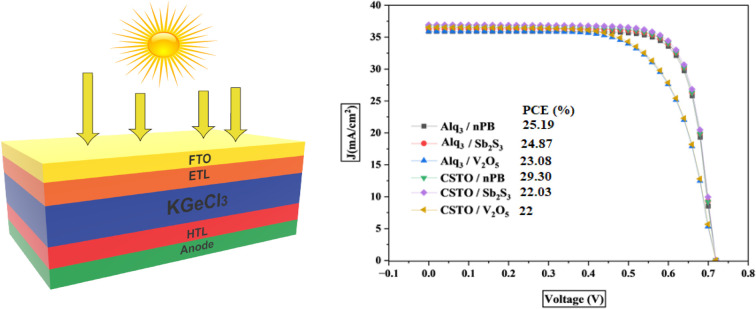

## 1. Introduction

Potassium germanium chloride (KGeCl_3_) has emerged as a novel perovskite material for use in photovoltaic applications, due to its unique optoelectronic properties and enhanced stability compared to traditional organic cation-based perovskites.^1^ In KGeCl_3_, potassium (K) is the A-site monovalent cation, germanium (Ge) the B-site divalent cation, and chloride (Cl) the X-site anion, forming a stable ABX_3_ crystal structure. The structure offers high thermal tolerance, withstanding temperatures exceeding 100 °C, and resistance to degradation from environmental exposure in comparison to the organic MA-based PSCs that degrade at around 60 °C.^2^ This positions KGeCl_3_ as a promising candidate for solar cell applications where longevity is critical. Unlike organic cation-based perovskites that are vulnerable to decomposition under moisture, heat, and UV exposure, KGeCl_3_ maintains structural integrity under these conditions, which could lead to more reliable and long-lasting photovoltaic devices.

KGeCl_3_-based perovskites exhibit strong light absorption within the visible spectrum, with an absorption coefficient exceeding 10^5^ cm^−1^, enhancing their suitability as absorber layers in perovskite solar cells. The material's optical bandgap of approximately 1.1 eV, along with low Auger recombination rates of less than 10^−8^ cm^3^ s^−1^, leads to efficient charge generation and minimizes energy losses.^3^ Furthermore, KGeCl_3_ has shown favorable charge carrier mobility, estimated to be in the range of 60–100 cm^2^ V^−1^ s^−1^, which produces rapid charge transport through the absorber layer and reduces recombination leading to enhanced overall power conversion efficiency (PCE).^4^ These characteristics make KGeCl_3_ a viable choice for PSCs, especially in contexts where environmental resilience and stability are prioritized over performance alone. Recent studies have demonstrated that KGeCl_3_-based PSCs can achieve competitive PCE values. Yasin *et al.* modelled a KGeCl_3_ based PSC in SCAPS with IGZO as the ETL.^5^ The cell produced competitive performance and achieved a PCE of 21.23% after thickness and doping optimization. In another study by Rehman *et al.*, they designed a PSC structure using KGeCl_3_ as the absorber with WS_2_ as the ETL and MoO_3_ as the HTL.^6^ Through design optimization including defect density, *R*_s_, *R*_sh_, and operating temperature the cell achieved a PCE of 29.02%.

One significant approach to enhancing the performance of Cs-PSCs is the careful selection of compatible charge transport layers (CTLs), which are essential for efficient charge transfer between the perovskite absorber and the electrodes.^7^ CTLs are classified into two types: hole transport layers (HTLs) and electron transport layers (ETLs).^8^ In the structure of a PSC, the HTL is at the back of the perovskite layer, facilitating hole extraction, while the ETL is placed at the front, aiding electron extraction. Perovskite materials are sandwiched between these layers. The use of conductive CTL materials with low resistance and proper band alignment can significantly improve the PCE of the PSCs. The effectiveness of these layers in charge transport directly impacts device efficiency by minimizing recombination losses and ensuring better energy band alignment. Alshomrany *et al.* used an Ag-AZO co-doped version of ZnO as an ETL in MAPbI_3_-based PSCs, which exhibited a significant increase in PCE to 27.26% compared to 25.98% achieved by its undoped counterpart.^9^ This enhancement is attributed to improved charge carrier extraction of the doped ETL. Similarly, Rasheed *et al.* showed that Rb-doped PSCs improved the PCE to 16.30% when the HTL was changed to CuAlO_2_ from NiO.^10^ This highlights the importance of selecting suitable CTL in achieving reliable and efficient PSC operation.

Research has shown that different combinations of CTL with specific perovskite absorbers can yield varying results, underscoring the importance of selecting the optimal combination for each perovskite material.^11^ This is largely because each perovskite material possesses a unique bandgap and electron affinity that affects the energy band alignment between the perovskite and the CTLs. Proper band alignment ensures effective charge extraction and transport, thereby reducing recombination rates and increasing the quantum efficiency of the cell.^12^ Moreover, this alignment influences the electric fields generated within the cell, which directly affects the charge separation and conductive potential of the device, all of which contribute to the overall performance of the PSC. Jan *et al.* explored multiple perovskite materials including FAPbI_3_, MAGeI_3_ and MASnI_3_ with different CTL of kesterites and zinc. They found that the most suitable CTL for FAPbI_3_ are CdZnS/CMTS (PCE 22.05%), for MAGeI_3_ are ZnO/CZTS (PCE 17.28%) and ZnO/CBTS for MASnI_3_ (PCE of 24.17%).^11^

Alq_3_ (tris(8-hydroxyquinolinato)aluminum) and CSTO (cadmium sulfide-titanium oxide) stand out as emerging ETLs with properties that are required by PSCs.^13,14^ Abbasi *et al.* achieved a PCE of 28.16% by using Alq_3_ as ETL with MASnI_3_ while Mahmood *et al.* achieved a PCE of 28.56% by using CSTO as ETL with MAPbI_3_.^13,14^ Alq_3_'s high charge mobility and CSTO's excellent chemical stability offer a robust framework for charge extraction and transport, essential in KGeCl_3_-based PSCs. Additionally, these materials exhibit good band energies, promoting seamless electron transfer by minimizing potential energy barriers. For HTLs, V_2_O_5_ (vanadium pentoxide), *n*PB (*N*,*N*′-di(1-naphthyl)-*N*,*N*′-diphenylbenzidine), and Sb_2_S_3_ (antimony sulfide) demonstrate favorable characteristics.^15,16^ Kuddus *et al.* used V_2_O_5_ as HTL in thin film solar cells and achieved a PCE of 23.5% after conducting design optimization while Basak *et al.* used Sb_2_S_3_ with the same cell and achieved a PCE of 12.62% without optimization. V_2_O_5_ is notable for its strong hole mobility, and *n*PB provides excellent conductivity, reducing the likelihood of charge accumulation within the cell. Sb_2_S_3_ enhances device stability under operational conditions, contributing to continuous charge transfer and reducing recombination rates. These HTLs support the efficient extraction of holes from the absorber layer, crucial for minimizing energy losses and enhancing PSC stability. Through selecting and integrating these five CTLs with KGeCl_3_, this study explores optimized charge transport and band alignment properties in PSCs. The band alignment and charge mobility of each CTL were rigorously evaluated through simulations, providing insight into their potential to improve power conversion efficiency.

In this study, the compatibility of KGeCl_3_ perovskite absorbers are investigated for the first time in with five novel CTLs (Alq_3_, CSTO, V_2_O_5_, *n*PB, Sb_2_S_3_), selected for their promising characteristics in improving charge transport and reducing recombination. The novelty of this work lies in the detailed exploration of the heterojunction compatibility between KGeCl_3_ and these CTLs, providing the first comprehensive insight into their band alignment, band offsets, and charge transfer mechanisms. This study not only evaluates the compatibility of these materials but also determines, for the first time, the optimized design parameters, such as layer thickness, doping concentrations, and defect densities, for these specific combinations. SCAPS software was used to perform numerical simulations of six different PSC structures, evaluating the impact of each CTL on the overall performance of the solar cell for the first time. Through these simulations, a systematic approach was applied to analyze the charge carrier dynamics and the electrostatic potential within the device. This method allowed for fine-tuning the cell design parameters to optimize device performance. Several critical factors were investigated to maximize the efficiency of the PSCs. These included the selection of CTLs, band alignment between the CTLs and the perovskite absorbers, band offsets, *I*–*V* characteristics, electric potential distribution within the cell, recombination rates, thickness, and doping levels of each layer. Additionally, the impact of temperature on device performance of each structure was carefully analyzed. The findings from this study not only establish optimized PSC designs for KGeCl_3_ and specific CTL combinations but also set a benchmark for further exploration of cesium-based perovskites in next-generation photovoltaics, emphasizing their potential for high efficiency, stability, and scalability.

## 2. Modelling

**Fig. 1:**
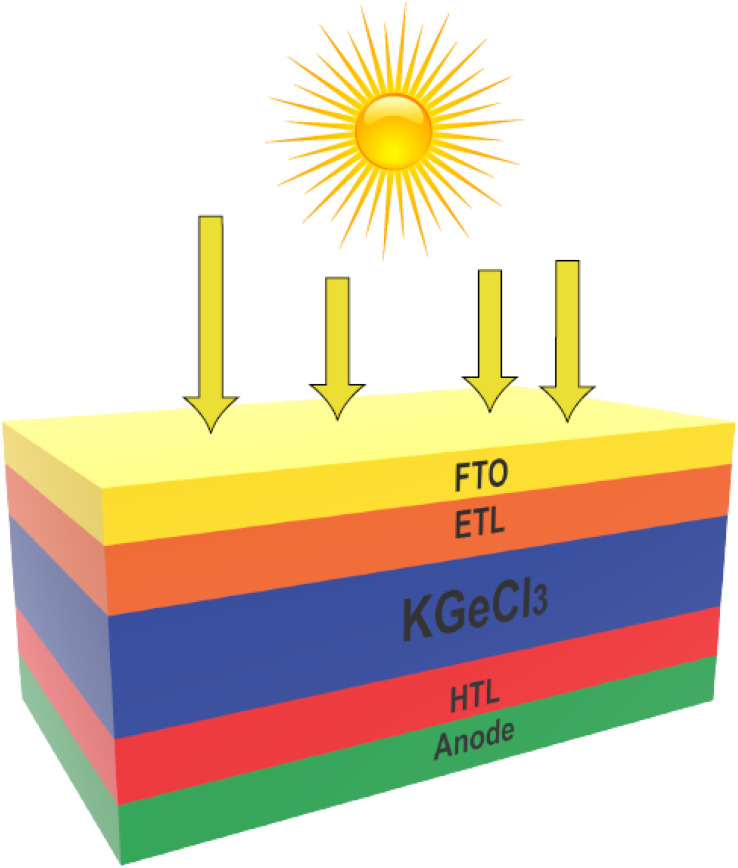
PSC structure.

In this study, a planar n-i-p configuration was selected for the KGeCl_3_-based PSCs. This structure has five distinct layers, with the ETL and HTL positioned around the KGeCl_3_ absorber. The ETLs, consisting of Alq_3_ and CSTO, are placed at the top of the absorber layer, while the HTLs, which include V_2_O_5_, *n*PB, and Sb_2_S_3_, are located at the bottom. The front electrode (cathode) and the back electrode (anode) are placed at either end of the device, completing the cell configuration. In this setup, the KGeCl_3_ perovskite layer absorbs photons, generating electron–hole pairs that are separated by the electric field at the heterojunctions formed with the CTLs.^17^ Electrons flow toward the ETLs and collected at the cathode, while holes move toward the HTLs and are collected at the anode.^18^ This cathode/ETL/KGeCl_3_/HTL/anode structure are formed by the PSCs which produce efficient charge separation and collection, thereby enhancing the overall power conversion efficiency of the PSCs. The final configuration of the device is illustrated in Fig. 1.

To evaluate the performance of the KGeCl_3_-based PSCs, simulations were conducted using the Solar Cell Capacitance Simulator (SCAPS) 1-D software. SCAPS solves essential photovoltaic equations, including the Poisson equation and continuity equations, which are instrumental in modeling the electrical behavior of the device.^19^ This simulation platform allows for precise adjustments to key design parameters across each layer of the PSC, such as layer thickness, doping, work function, bandgap energy, charge carrier mobility, electron affinity, permittivity, and doping density. These parameters are critical in understanding and optimizing the performance characteristics of PSCs with KGeCl_3_ as the absorber material.

To increase the accuracy of simulation results, defect layers were incorporated into both the bulk and interfacial regions of the PSC materials.^20^ The defect density in the bulk of the KGeCl_3_ absorber layer and CTLs was set at 1 × 10^15^ cm^−3^, while the interfacial defect density between the CTLs and the KGeCl_3_ layer was 1 × 10^14^ cm^−3^. These defect layers account for trap states and structural imperfections within the active layer, which can hinder the mobility of charge carriers.^21^ The interfacial defects, including grain boundaries, surface irregularities, and dangling bonds, were also considered to closely simulate real-world conditions.^22^ For electrode materials, the anode was modeled as gold (Au) with a work function of 5.1 eV, and the cathode as fluorine-doped tin oxide (FTO) glass with a work function of 4.4 eV. Table 1 details the specific design parameters used for each material in the PSC configuration. The table includes references to the relevant literature from which the values were sourced.^5–7,13–17^ Parameters such as bandgap, electron affinity, electric permittivity, and mobility are critical and remain fixed in the simulations. These have been directly taken from validated and reliable sources in literature. The referenced studies have derived these values from experimental results, ensuring that the simulations are grounded in realistic material properties.

**Table 1:** Material parameters used in SCAPS-1D

Parameters	KGeCl_3_ (ref. 5 and 7)	Alq_3_ (ref. 13)	CSTO^14^	V_2_O_5_ (ref. 15)	*n*PB^16^	Sb_2_S_3_ (ref. 17)
Thickness *w* (μm)	0.300	0.100	0.100	0.050	0.150	0.150
Band gap *E*_g_ (eV)	1.100	2.800	2.960	2.200	2.400	1.620
Electron affinity *χ* (eV)	4.000	4.170	3.900	3.400	3.000	3.700
Dielectric permittivity (*ε*_r_)	23.010	3.400	9.000	8.000	3.000	7.080
CB effective density of states (cm^−3^)	1 × 10^18^	1.440 × 10^20^	2.700 × 10^19^	9.200 × 10^19^	2.700 × 10^19^	2.000 × 10^19^
VB effective density of states (cm^−3^)	1 × 10^18^	1.440 × 10^20^	3.500 × 10^20^	5.000 × 10^20^	3.500 × 10^20^	1.000 × 10^19^
Electron mobility (cm^2^ V s^−1^)	9.292 × 10^1^	1.900 × 10^−5^	6.000 × 10^3^	1.000 × 10^7^	6.100	9.800
Hole mobility (cm^2^ V s^−1^)	6.859 × 10^1^	2.000 × 10^−7^	6.600 × 10^2^	1.000 × 10^7^	6.100	1.000 × 10^1^
Donor doping concentration (cm^−3^)	—	1.000 × 10^21^	1.000 × 10^17^	—	—	—
Acceptor doping concentration (cm^−3^)	1.000 × 10^15^	—	—	1.000 × 10^18^	1.000 × 10^18^	1.000 × 10^18^
Electron thermal velocity (cm s^−1^)	1.0 × 10^7^	1.0 × 10^7^	1.0 × 10^7^	1.0 × 10^7^	1.0 × 10^7^	1.0 × 10^7^
Hole thermal velocity (cm s^−1^)	1.0 × 10^7^	1.0 × 10^7^	1.0 × 10^7^	1.0 × 10^7^	1.0 × 10^7^	1.0 × 10^7^
Defect type	Neutral	Neutral	Neutral	Neutral	Neutral	Neutral
Energetic distribution	Gaussian	Neutral	Neutral	Neutral	Neutral	Neutral
Defect density *N*_t_ (cm^−3^)	10^15^	10^15^	10^15^	10^15^	10^15^	10^15^

The parameters such as layer thickness, doping densities, and defect densities are variable and were also initially taken from the literature for preliminary simulations. These values were then systematically varied within a practical range to identify realistic optimized values that maximize device performance. This approach ensures that the simulated results closely align with experimental conditions and produce meaningful insights into the performance of KGeCl_3_-based PSCs. By combining fixed values from validated literature and optimized variable parameters, this study employs a robust and scientifically justified methodology to ensure the accuracy and reliability of the SCAPS-1D simulation results.

## 3. Layer compatibility

### 3.1. Band alignment

In PSCs, the alignment of energy bands between the KGeCl_3_ absorber and the CTLs is a key factor that influences charge separation and transport efficiency. For efficient charge movement, electrons should flow from the perovskite to the ETL through the conduction band, while holes should flow from the perovskite to the HTL through the valence band.^23^ The degree of alignment, or band offset, between these layers is crucial for effective carrier transport and separation. For optimal compatibility between KGeCl_3_ and the ETLs, the conduction band offset (CBO) should be minimized to enable smooth electron flow across the interface. A minimal CBO reduces the energy barrier, allowing electrons to move efficiently into the ETL. While the valence band offset (VBO) should be maximized to prevent hole movement into the ETL, thereby reducing recombination losses.^24^ This selective transport of electrons ensures high charge separation efficiency and enhances device performance.

Similarly, in the HTL, a low VBO enables effective hole transfer from KGeCl_3_, while a high CBO acts as a barrier to electron backflow, maintaining selective charge transport toward the anode. Proper alignment between the valence and conduction bands of the HTL and KGeCl_3_ absorber is thus essential for promoting efficient charge extraction and minimizing carrier recombination. Table 2 shows the CBO and VBO for each CTL used with the KGeCl_3_ absorber layer. The CBO and VBO for all the perovskite/CTLs interfaces calculated through eqn (1) & (2).^25^

1 CBO = *χ*_Per_ − *χ*_CTL_

2 VBO = *χ*_CTL_ −*χ*_Per_ + Eg_CTL_ − Eg_Per_

Eg_CTL_ and Eg_Per_ denote band gap energy of perovskite and CTL, respectively while χ_Per_ and χ_CTL_ represents electron affinity of perovskite and CTL, respectively.^26^

**Table 2:** Band Offsets between the perovskite and CTL

CTL	CBO	VBO
Alq_3_	−0.17	1.87
CSTO	0.1	1.76
V_2_O_5_	0.7	0.4
*n*PB	1	0.3
Sb_2_S_3_	0.3	0.22

Among the ETLs, Alq_3_ produces a CBO of −0.17 eV, forming a slight cliff. The electrons face a small energy barrier when moving from the KGeCl_3_ layer to the Alq_3_ ETL. However, this minimal cliff is less likely to significantly hinder electron transport. The VBO of 1.87 eV provides effective hole-blocking, preventing holes from entering the ETL and minimizing recombination losses. The small CBO combined with a high VBO makes Alq_3_ a promising ETL for KGeCl_3_ in promoting selective electron extraction. Similarly, the CSTO has a CBO of 0.1 eV, forming a small spike that slightly favors electron transfer from KGeCl_3_ to the ETL by reducing the barrier at the interface. Its VBO of 1.76 eV further enhances hole-blocking capabilities, which is beneficial for maintaining charge separation. This balance between minimal electron-blocking and strong hole-blocking abilities makes CSTO a compatible ETL with KGeCl_3_, supporting efficient electron extraction and retention.

For the HTLs, V_2_O_5_ produces a CBO of 0.7 eV that effectively prevents electron flow, thereby enhancing electron-blocking characteristics. Its VBO of 0.4 eV indicates a barrier for holes, which may hinder hole extraction. This combination of moderate CBO and VBO makes V_2_O_5_ a HTL option. The *n*PB, with a CBO of 1 eV, establishes a substantial electron-blocking barrier, which effectively prevents electrons from flowing into the HTL and thus reduces recombination risk. Its VBO of 0.3 eV provides a lower barrier for hole transfer, facilitating efficient hole transport from the KGeCl_3_ absorber to the HTL. This configuration of high electron-blocking and low hole-blocking properties positions *n*PB as a strong HTL candidate for use with KGeCl_3_ in PSCs. Lastly, Sb_2_S_3_ produces a CBO of 0.3 eV, forming a moderate barrier that prevents electron flow while still maintaining adequate compatibility with KGeCl_3_. The VBO of 0.22 eV further supports hole transport by offering minimal resistance, allowing holes to transfer efficiently. This combination of moderate CBO and minimal VBO suggests that Sb_2_S_3_ could also serve as an effective HTL for KGeCl_3_, contributing to efficient charge extraction.

### 3.2. Electric potential & recombination at hetero-junction

The electric potential at the heterojunctions between the KGeCl_3_ and the CTLs plays a pivotal role in determining the efficiency of charge separation and transport within the PSC. This potential is influenced directly by the conduction band offset (CBO) and valence band offset (VBO) established between the CTL and KGeCl_3_.^27^ The electric potential generated at the heterojunction facilitates the separation of photogenerated electron–hole pairs by creating a built-in electric field, which drives electrons toward the ETL and holes toward the HTL.^28^ The magnitude of this potential is critically dependent on the band offsets at the CTL/KGeCl_3_ interface.

A positive band offset, or “spike,” at the heterojunction generally increases the electric potential barrier, increasing the flow of charge carriers to the CTL.^29^ Moderate spikes help suppress charge carrier recombination by preventing the backflow of undesired carriers (*e.g.*, holes in ETL or electrons in the HTL). For example, CSTO, with a CBO of 0.1 eV, forms a slight spike that enhances electron transport while simultaneously blocking hole backflow. Similarly, V_2_O_5_ produces a substantial CBO of 0.7 eV, effectively blocking electrons from the HTL, which is essential for reducing recombination rates.^28^ Such spikes can be beneficial at moderate levels as they minimize recombination. However, large spikes can restrict the desired charge carriers' movement, creating internal resistance, reducing current density, and impacting the overall PCE of the PSC.

**Fig. 2:**
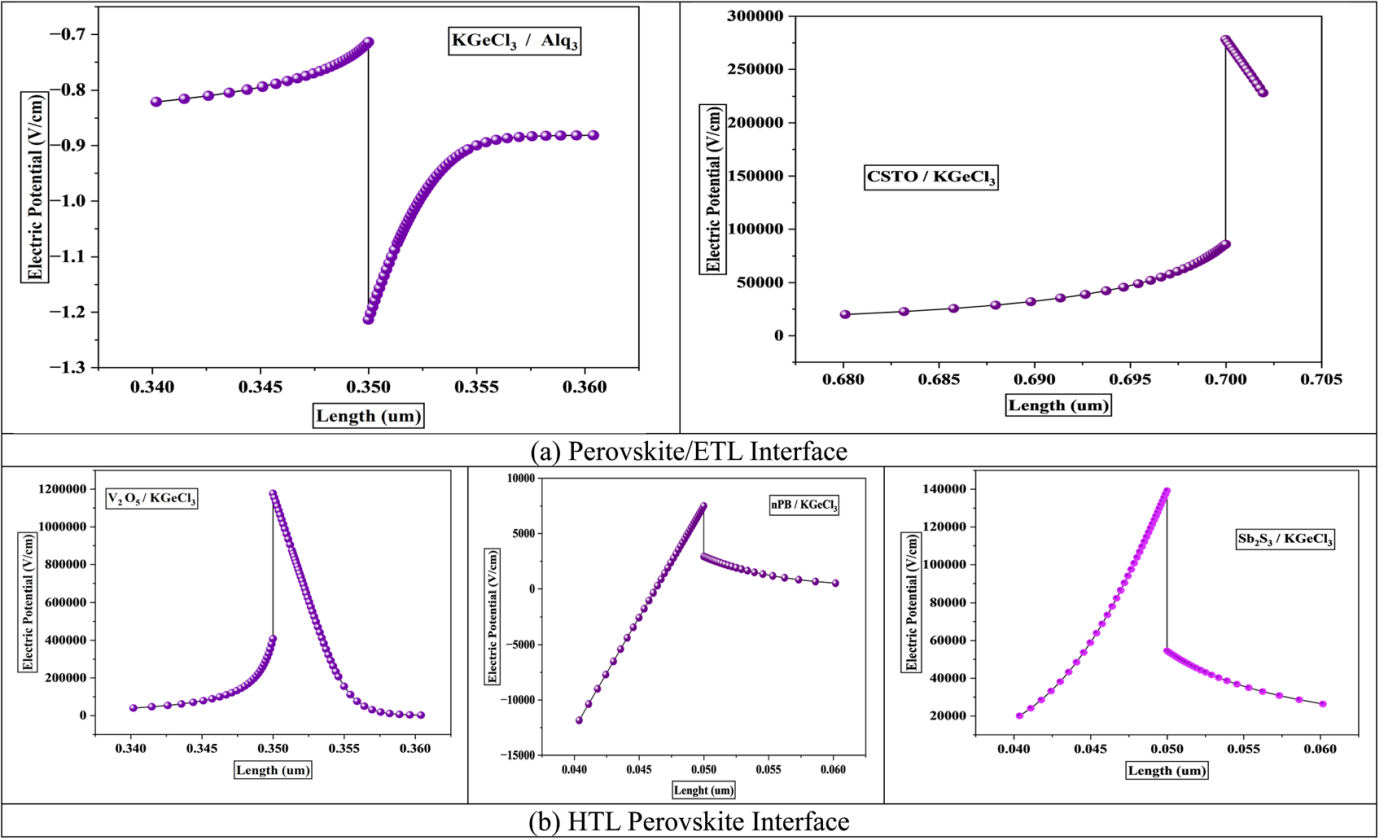
Electric field at interface.

Conversely, a negative band offset, or “cliff,” tends to lower the electric potential barrier at the heterojunction. For instance, Alq_3_, with a CBO of −0.17 eV, creates a slight cliff at the KGeCl_3_/ETL interface.^29^ This reduced potential can decrease charge extraction efficiency and increases the likelihood of recombination at the heterojunction. This recombination rates, which can reduce carrier lifetimes and quantum efficiency. Fig. 2 shows the electric potential across the CTL/KGeCl_3_ heterojunction for different CTL combinations. The figure illustrates how spikes in band alignment contribute to higher electric potential, improving charge carrier separation by enhancing the driving force for electrons and holes. On the other hand, cliffs reduce this potential, leading to a weaker built-in electric field and higher recombination rates.^30^ Understanding this balance is essential for optimizing the CTL/KGeCl_3_ interface for maximum device performance.

The band offsets and the resulting electric potential at the heterojunction significantly influence recombination rates at the interface between the CTLs and the KGeCl_3_ absorber layer. A large band offset, whether as a positive spike or a negative cliff, increases recombination at the heterojunction.^31^ This increase in recombination is due to the energy level mismatch by large offsets, which trap charge carriers at the interface. For example, a significant spike in the conduction band can impede electron flow from the absorber to the ETL, while a large cliff may similarly hinder hole movement into the HTL. These trapped carriers remain at the interface for prolonged periods, increasing the likelihood of recombining with opposite carriers. This process reduces the overall power conversion efficiency (PCE) of the PSC.

The electric potential across the heterojunction is another key factor influencing recombination rates.^32^ Lower electric potential at the interface lacks the necessary driving force to influence and move charge carriers across the heterojunction barrier, leading to a higher recombination. Without sufficient potential, carriers tend to linger in the absorber, increasing the recombination. This lack of mobility results in significant losses in quantum efficiency and short-circuit current density.^31^ While higher electric potential assists in swiftly moving charge carriers across the interface, thereby reducing the time they spend at the heterojunction and lowering recombination chances.

**Fig. 3:**
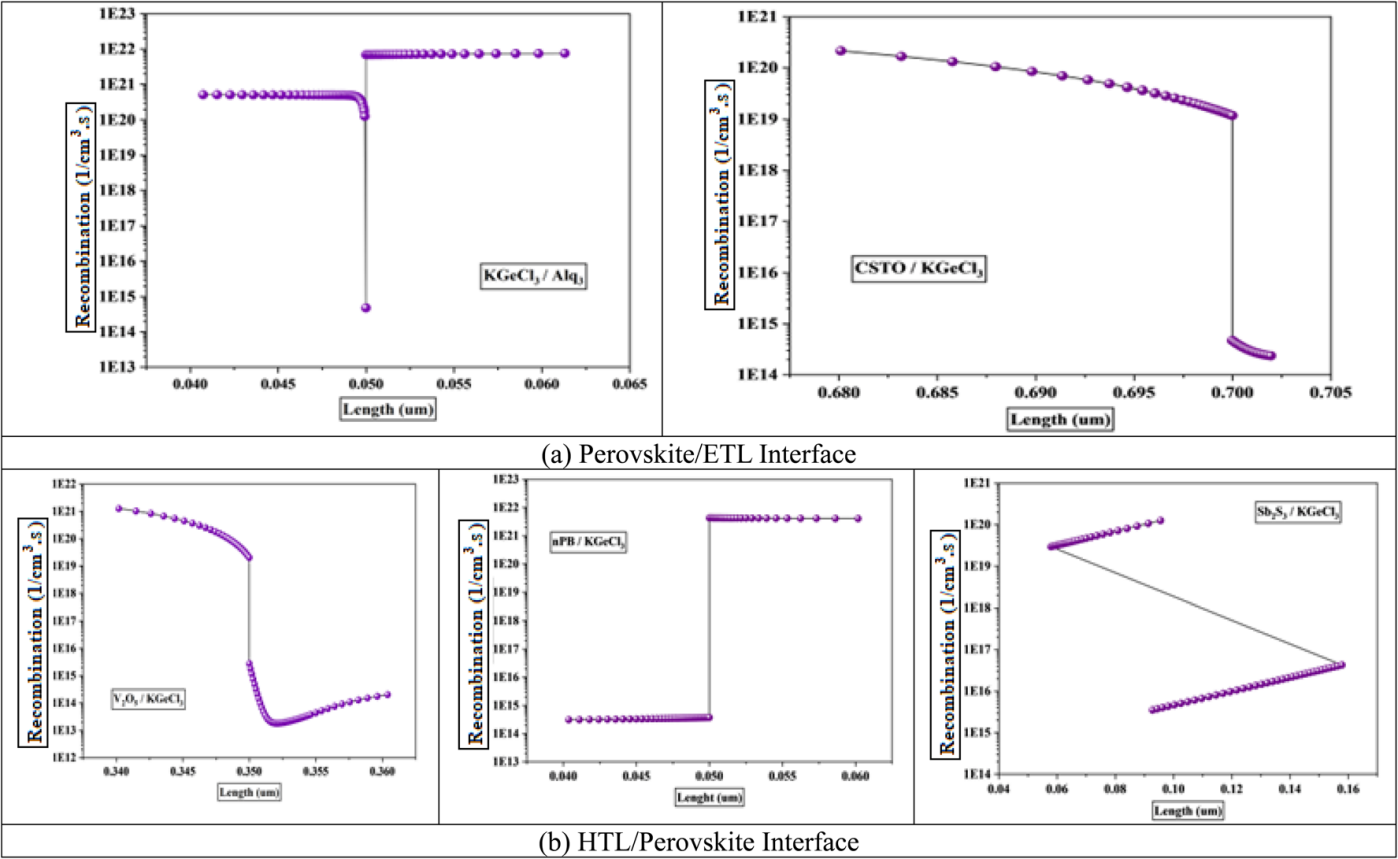
Recombination at interface.

Fig. 3 shows the recombination rates across different heterojunction configurations, highlighting the correlation between electric potential and recombination. As observed, lower electric potential produces significantly increased recombination rates, as the energy provided is insufficient for carriers to cross the heterojunction barrier efficiently. In contrast, a higher electric potential enhances carrier movement, minimizing recombination by expediting their transit across the heterojunction before interaction with opposite carriers can occur.

### 3.3. *I*–*V* results

**Fig. 4:**
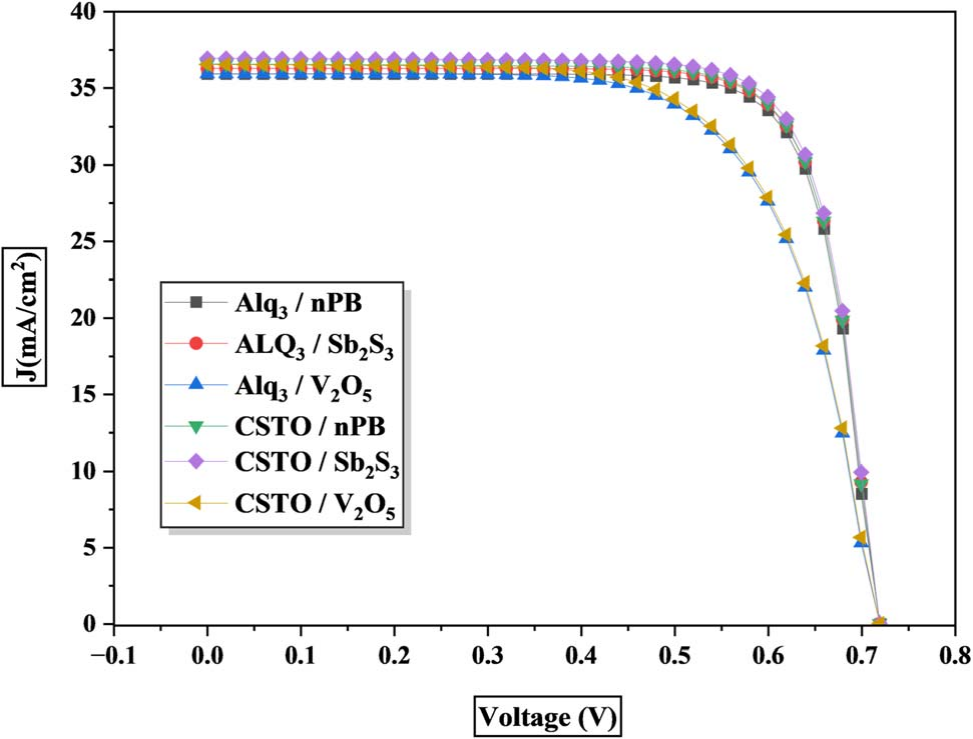
*I*–*V* curves of the PSCs.

In this study, six different PSC configurations were modeled by varying the ETLs and HTLs with the KGeCl_3_ perovskite absorber. The performance of each PSC structure was evaluated by analyzing the short-circuit current density (*J*_sc_), open-circuit voltage (*V*_oc_), fill factor (FF), and overall power conversion efficiency (PCE). These parameters are critical for assessing the overall performance of PSCs, as they reflect the efficiency of charge carrier generation, separation, and transport within the device. Table 3 summarizes the results, while the detailed *I*–*V* curves for each configuration are presented in Fig. 4.

**Table 3:** *I*–*V* analysis of the PSCs

S. No.	Structures	*J*_sc_ (mA cm^−2^)	*V*_oc_ (V)	FF	%
1	Alq_3_-KGeCl_3_-*n*PB	35.9256	0.711045	78.8908	20.1393
2	Alq_3_-KGeCl_3_-Sb_2_S_3_	36.2886	0.711908	78.9196	20.3777
3	Alq_3_-KGeCl_3_-V_2_O_5_	35.9240	0.711732	68.128	17.4192
4	CSTO-KGeCl_3_-Sb_2_S_3_	36.76273	0.716275	78.9013	20.7765
5	CSTO-KGeCl_3_-*n*PB	36.5711	0.711397	78.491	20.4207
6	CSTO-KGeCl_3_-V_2_O_5_	36.5438	0.712535	67.4854	17.5723

The first set of PSCs used Alq_3_ as the ETL with KGeCl_3_. The Alq_3_-KGeCl_3_-*n*PB configuration achieved a *J*_sc_ of 35.93 mA cm^−2^, a *V*_oc_ of 0.71 V, and an FF of 78.89%, resulting in a PCE of 20.14%. The high efficiency is due to the good compatibility between Alq_3_ and *n*PB as CTLs with KGeCl_3_, supporting effective charge extraction and minimal recombination. A slight improvement was observed when Sb_2_S_3_ was used as the HTL, which produced a PCE of 20.38%. This increase in efficiency is because Sb_2_S_3_ provides better band alignment with KGeCl_3_ than *n*PB, resulting in improved charge transport. However, when V_2_O_5_ was used as the HTL, the FF dropped significantly to 68.13%, resulting in a lower PCE of 17.42%. This decrease in performance is due to the less optimal band alignment between V_2_O_5_ and KGeCl_3_, leading to higher recombination rates and less efficient charge extraction.

The second set of configurations use CSTO as the ETL. The CSTO-KGeCl_3_-Sb_2_S_3_ configuration demonstrated the highest performance, achieving a *J*_sc_ of 36.76 mA cm^−2^, a *V*_oc_ of 0.72 V, an FF of 78.90%, and a PCE of 20.78%. The superior performance of this configuration is due to the excellent compatibility of CSTO and Sb_2_S_3_ with KGeCl_3_, enabling efficient charge separation and transport. When *n*PB was used as the HTL, the PCE was slightly lower at 20.42%. This reduction can be linked to minor differences in band alignment, as *n*PB exhibits slightly higher VBO compared to Sb_2_S_3_, which may introduce additional resistance during hole transport. In the CSTO-KGeCl_3_-V_2_O_5_ configuration, the FF dropped to 67.49%, resulting in a reduced PCE of 17.57%, due to the poor band alignment between V_2_O_5_ and KGeCl_3_. The significant barrier for hole transfer and increased recombination at the interface contribute to the lower performance.

## 4. Optimization

### 4.1. Layer thickness optimization

The thickness of each layer in the KGeCl_3_-based PSCs is a critical factor influencing the device's overall performance. In this study, the thicknesses of the KGeCl_3_ absorber layer and the charge transport layers (ETL and HTL) were systematically varied to study their effects on parameters such as *J*_sc_, *V*_oc_, FF, and PCE. Layer thickness optimization is essential for balancing photon absorption and charge transport efficiency, ensuring that the device operates at its maximum potential.^33^ These optimizations provided valuable insights into the impact of layer thickness on key performance metrics.

**Fig. 5:**
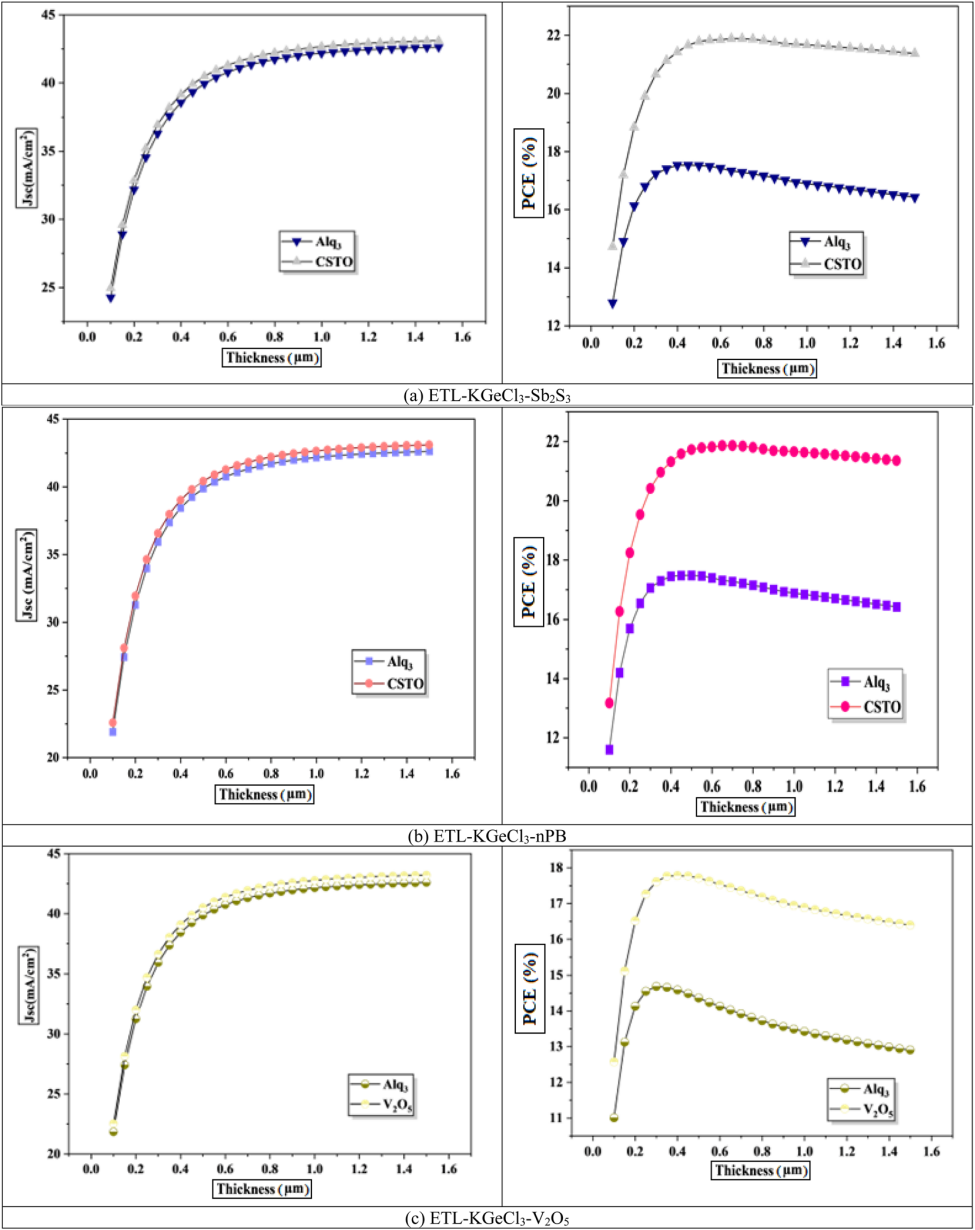
Effect of absorber thickness.

The initial focus was on optimizing the thickness of the KGeCl_3_ absorber layer, with results presented in Fig. 5. As the absorber layer's thickness increased, there was a corresponding rise in *J*_sc_, which led to an improvement in the PCE. This increase is due to enhanced photon absorption in a thicker absorber layer, which generates a higher photocurrent and, therefore, a greater *J*_sc_.^34^ However, after reaching an optimal thickness, further increases resulted in a decline in performance. Beyond this optimal point, the additional thickness increases the carrier path length, which in turn raises the chances of recombination due to longer carrier lifetimes.^33^ This trade-off between photon absorption and carrier recombination highlights the importance of carefully optimizing the absorber layer thickness to achieve maximal device efficiency without compromising charge transport.

The results further show that the choice of CTL combination has a major influence on the optimized thickness of the KGeCl_3_ absorber layer. CTLs with superior band alignment produce a thicker absorber layer to achieve optimal performance. This is because a well-aligned band structure produces a stronger electric field across the heterojunction, allowing it to extend deeper into the absorber.^28^ This enhanced field assists in effectively separating charge carriers over a larger region within the absorber, thus supporting higher *J*_sc_ and improved overall efficiency.

While CTLs with less favorable band alignment produces thinner optimized absorber layers. This is because increased thickness can exacerbate recombination and hinder charge extraction efficiency. In such cases, a thinner absorber helps to reduce carrier path lengths, thereby minimizing recombination losses. This correlation between band alignment and optimized absorber thickness underscores the importance of selecting CTLs that not only align well with the absorber but also facilitate efficient charge separation, enhancing PSC performance.

**Fig. 6:**
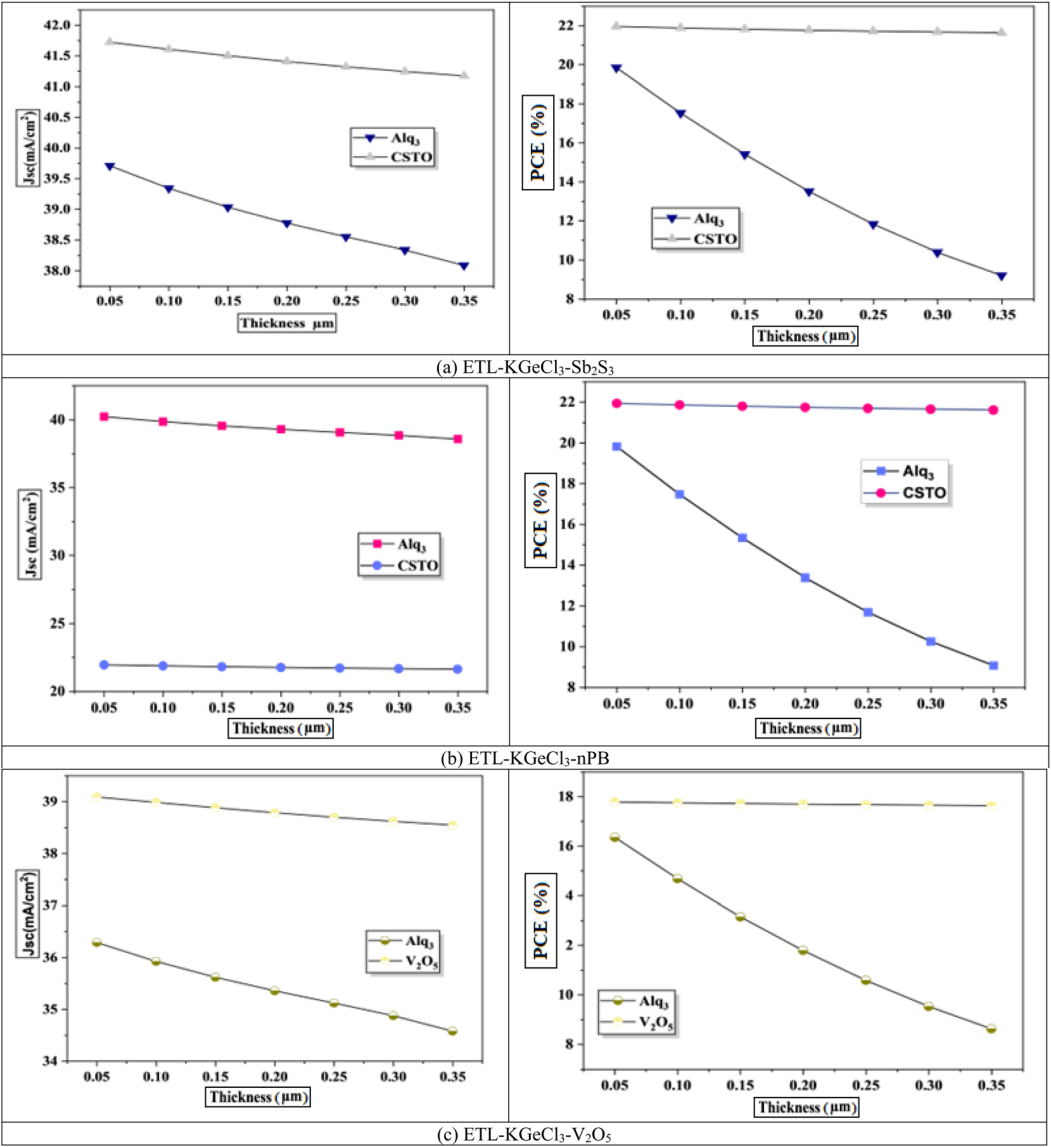
Effect of ETL thickness.

**Fig. 7:**
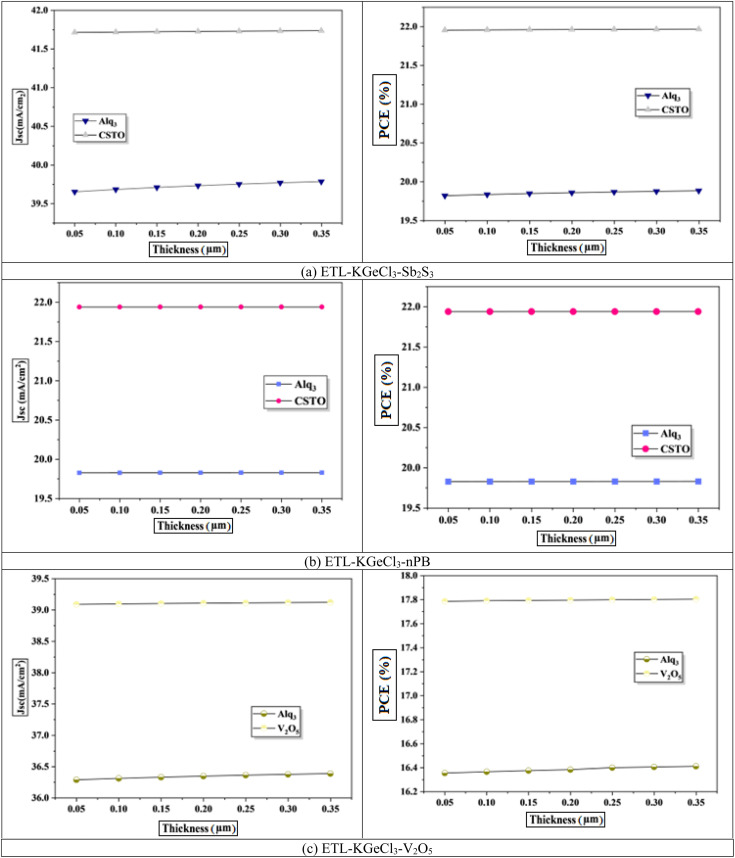
Effect of HTL thickness.

The impact of CTL thickness on PSC performance was also investigated, as shown in Fig. 6 and 7. Layer thickness plays a significant role in determining the efficiency of light absorption, charge transport, and overall device performance, particularly for the ETL and HTL, which directly interact with the KGeCl_3_ absorber.^35^ Increasing the thickness of the ETL leads to a decline in device efficiency (Fig. 6). A thicker ETL can absorb a portion of the incident light, reducing the light intensity reaching the KGeCl_3_ absorber layer, which in turn decreases the generation of charge carriers.^36^ Additionally, a thicker ETL introduces higher series resistance within the device, impeding electron flow and thereby reducing the overall PCE.

In contrast, variations in the HTL thickness exhibit a comparatively milder effect on performance. While increasing the HTL thickness does slightly raise the series resistance, this effect is less detrimental to the device's performance than the impact of a thicker ETL. HTL primarily facilitates hole transport, and as long as its thickness remains within a reasonable range, charge carriers can still be efficiently extracted.^25^ However, excessively thick HTLs may lead to a slight reduction in *V*_oc_ and fill factor due to increased resistance.^37^ Thus, while optimizing HTL thickness is still important, it is less critical than achieving an optimal ETL thickness, which has a more direct influence on light absorption and charge transport within the PSC.

The final optimized thickness of the absorber for each PSC structure is summarized in Table 4, along with the improved performance metrics. For all configurations, the optimized ETL and HTL thicknesses were set to 100 nm. This value ensures a balance between minimal resistance and sufficient optical and electrical properties to support efficient charge extraction.

**Table 4:** Optimized parameters and results

S. No.	Structures	Absorber thickness (μm)	Absorber doping (cm^−3^)	ETL doping (cm^−3^)	*J*_sc_ (mA cm^−2^)	*V*_oc_ (V)	FF	%
1	Alq_3_-KGeCl_3_-*n*PB	500	1 × 10^17^	1 × 10^19^	40.235	0.75520	82.9125	25.1938
2	Alq_3_-KGeCl_3_-Sb_2_S_3_	450	1 × 10^17^	1 × 10^19^	39.648	0.75696	82.8808	24.8744
3	Alq_3_-KGeCl_3_-V_2_O_5_	300	1 × 10^17^	1 × 10^19^	36.762	0.76358	82.2518	23.0892
4	CSTO-KGeCl_3_-Sb_2_S_3_	650	1 × 10^14^	1 × 10^18^	41.826	0.67419	78.1512	22.0381
5	CSTO-KGeCl_3_-*n*PB	650	1 × 10^18^	1 × 10^17^	41.804	0.81522	85.9786	29.302
6	CSTO-KGeCl_3_-V_2_O_5_	400	1 × 10^16^	1 × 10^19^	39.032	0.71194	79.1734	22.0012

### 4.2. Layer doping optimization

Next optimizing the doping concentration of the KGeCl_3_ absorber layer was done. As an intrinsic semiconductor, KGeCl_3_'s optoelectronic properties can be adjusted by introducing dopants into the material.^38^ Increasing the doping concentration in a semiconductor impacts key properties, including carrier concentration, *V*_oc_, recombination rate, and diffusion length, all of which influence device performance. This optimization step aims to strike a balance between enhancing charge carrier mobility and concentration while maintaining the semiconductor characteristics of KGeCl_3_.^39^

**Fig. 8:**
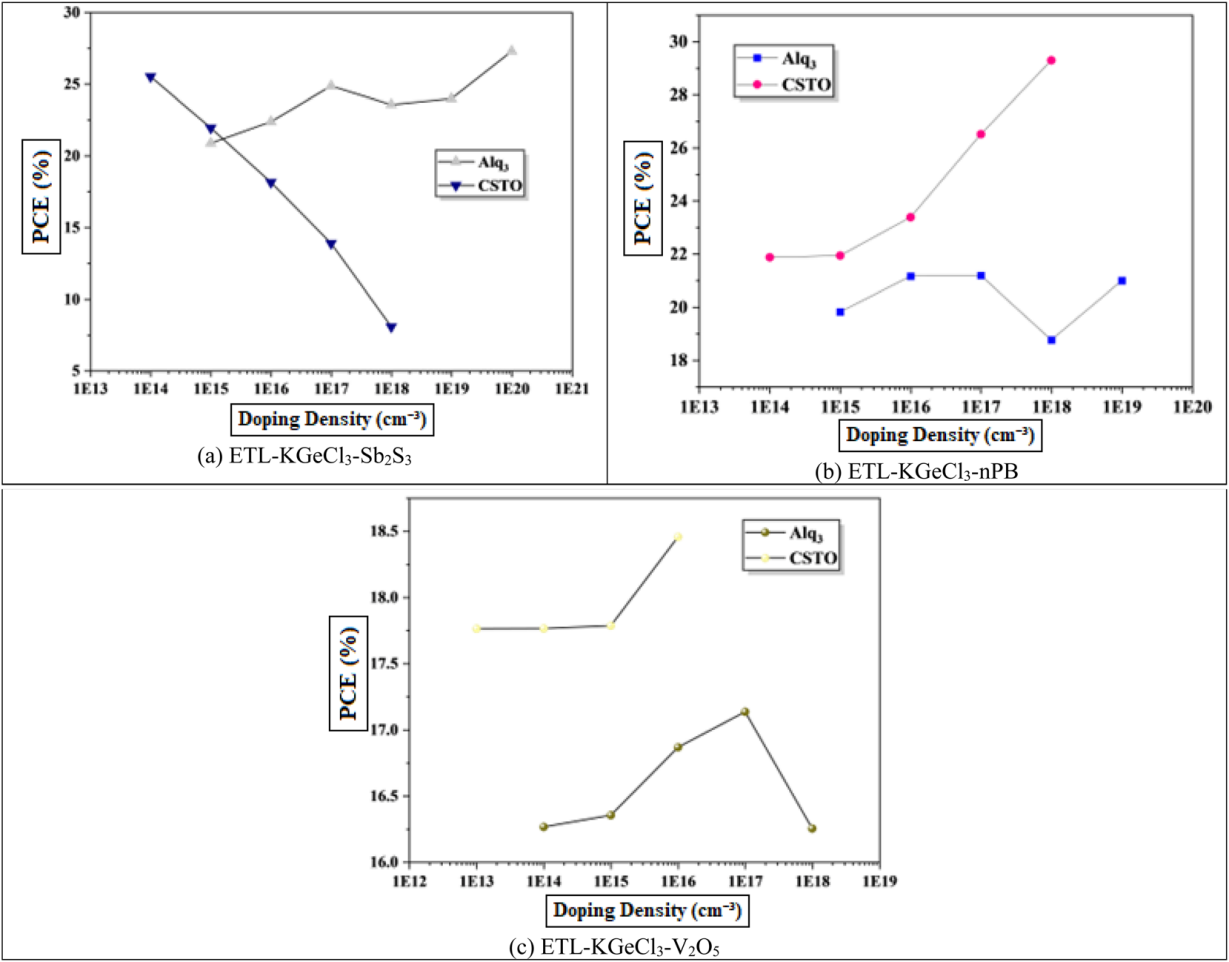
Effect of absorber doping.

The perovskite materials doping offers flexibility to tune between n-type and p-type properties by adjusting the ratios of precursor materials during fabrication. This inherent tunability is an advantage in PSC design, as it allows precise control over the absorber's electronic behavior to complement the charge transport layers.^39^ This study focuses on enhancing p-type doping as shown in Fig. 8. The results show that increasing doping levels increase carrier mobility and concentration, thereby improving electron and hole transport up to an optimal point.^38^ This enhanced transport reduces recombination losses and increases *V*_oc_, which contributes to higher device performance. Beyond this point, however, further increases in doping concentration compromise the semiconductor nature of KGeCl_3_, introducing metallic properties that reduce device efficiency due to hindered charge separation and transport. Excessively high doping concentrations lead to a shift from semiconductor to metallic behavior, which hampers charge transport. In such cases, the diffusion length of carriers is reduced, and recombination rates increase due to the high density of free carriers. This transition from semiconductor to metallic behavior results in a decline in *V*_oc_ and PCE, as the material's ability to maintain an internal electric field is significantly reduced.^20^ The optimized doping concentrations determined in this study are presented in Table 4.

To further enhance PSC performance, the CTLs doping was conducted to improve conductivity and charge separation efficiency.^40^ Doping plays a crucial role in modifying the electronic properties of the CTLs, enhancing their ability to extract and transport charge carriers efficiently. In this study, high acceptor doping (*N*_A_) was introduced in the. The high doping level in the HTL reduces its resistance and increases its conduction which facilitates efficient hole extraction and transport from the KGeCl_3_ absorber to the anode. A well-doped HTL minimizes energy losses and ensures that holes are quickly and effectively collected, thereby reducing the likelihood of recombination losses at the absorber/HTL interface.^41^ Similarly, the ETL was doped with a high level of donor doping (*N*_D_).^42^ This high doping concentration in the ETL enhances electron conductivity, supporting effective electron extraction and transport to the cathode.

**Fig. 9:**
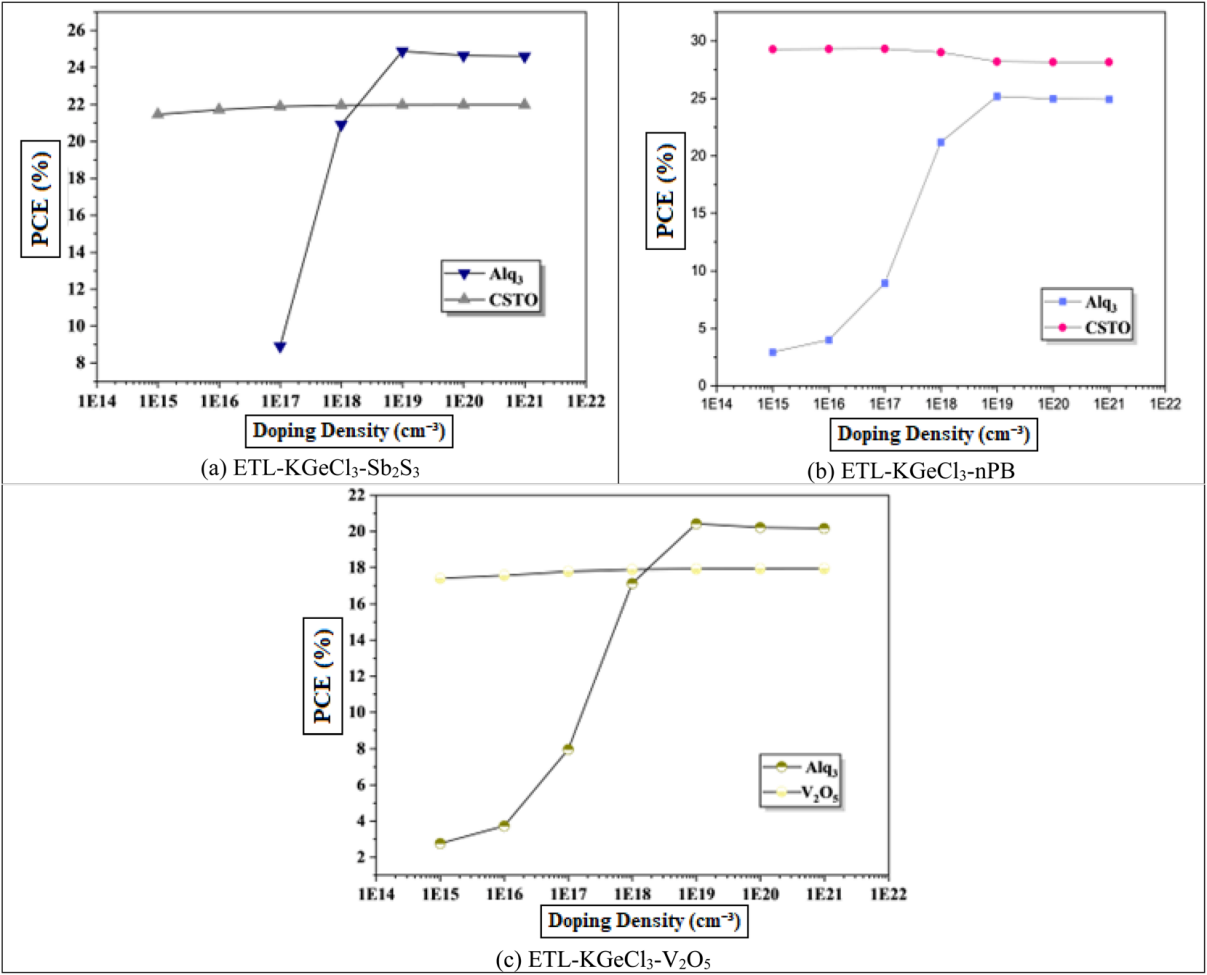
Effect of ETL doping.

**Fig. 10:**
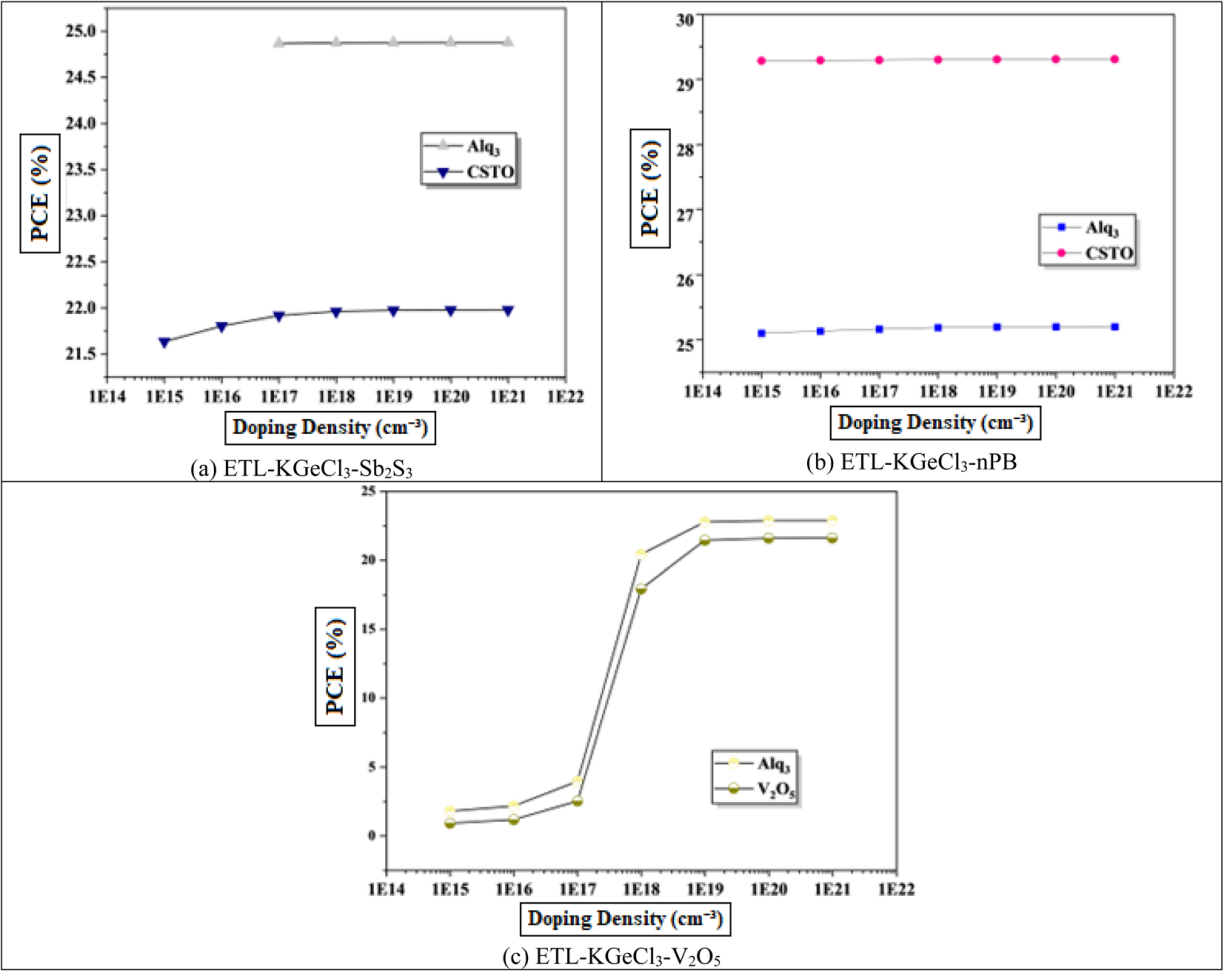
Effect of HTL doping.

The influence of varying doping concentrations in both ETL and HTL on PSC performance is shown in Fig. 9 and 10. As the doping levels in both CTLs increase, there is a significant improvement in device performance. This enhancement is primarily due to the increased conductivity of the CTLs, which enables more efficient charge collection and transport to the electrodes with minimal losses.^43^ Improved conductivity reduces the series resistance within the PSC, promoting more effective charge separation and decreasing recombination risks at the interfaces. Consequently, the PCE rises markedly with increased CTL doping levels. However, beyond an optimal doping concentration, no significant improvement is observed, as excessive doping leads to saturation of conductivity benefits. This saturation occurs because, after a certain point, the resistance within the CTL is already minimized, and further doping does not substantially enhance charge transport properties.^40^ Instead, it may increase the risk of interface defects due to the high density of charge carriers, which could adversely affect long-term stability.

Through extensive optimization, the ideal doping concentrations were identified, with an optimal doping level of 1 × 10^20^ cm^−3^ for all HTLs. The optimized doping concentrations for each ETL are detailed in Table 4.

### 4.3. Layer defects optimization

In PSCs, the carrier lifetime is inversely related to the total defect density; thus, an increase in defect density leads to a shorter charge carrier lifetime and a reduced diffusion length.^44^ The diffusion length is a critical parameter, as it determines how far charge carriers can travel before recombining. A shorter diffusion length means fewer carriers reach the electrodes, reducing the device's overall power output.^45^ This reduction adversely affects key performance parameters, including *V*_oc_, *J*_sc_, and overall PCE. Higher defect density introduces more structural imperfections, creating additional traps for charge carriers, which enhances recombination rates according to Shockley–Read–Hall (SRH) recombination theory.^46^ This increased recombination rate directly impacts the efficiency of the solar cell by reducing the number of charge carriers that contribute to current generation.

**Fig. 11:**
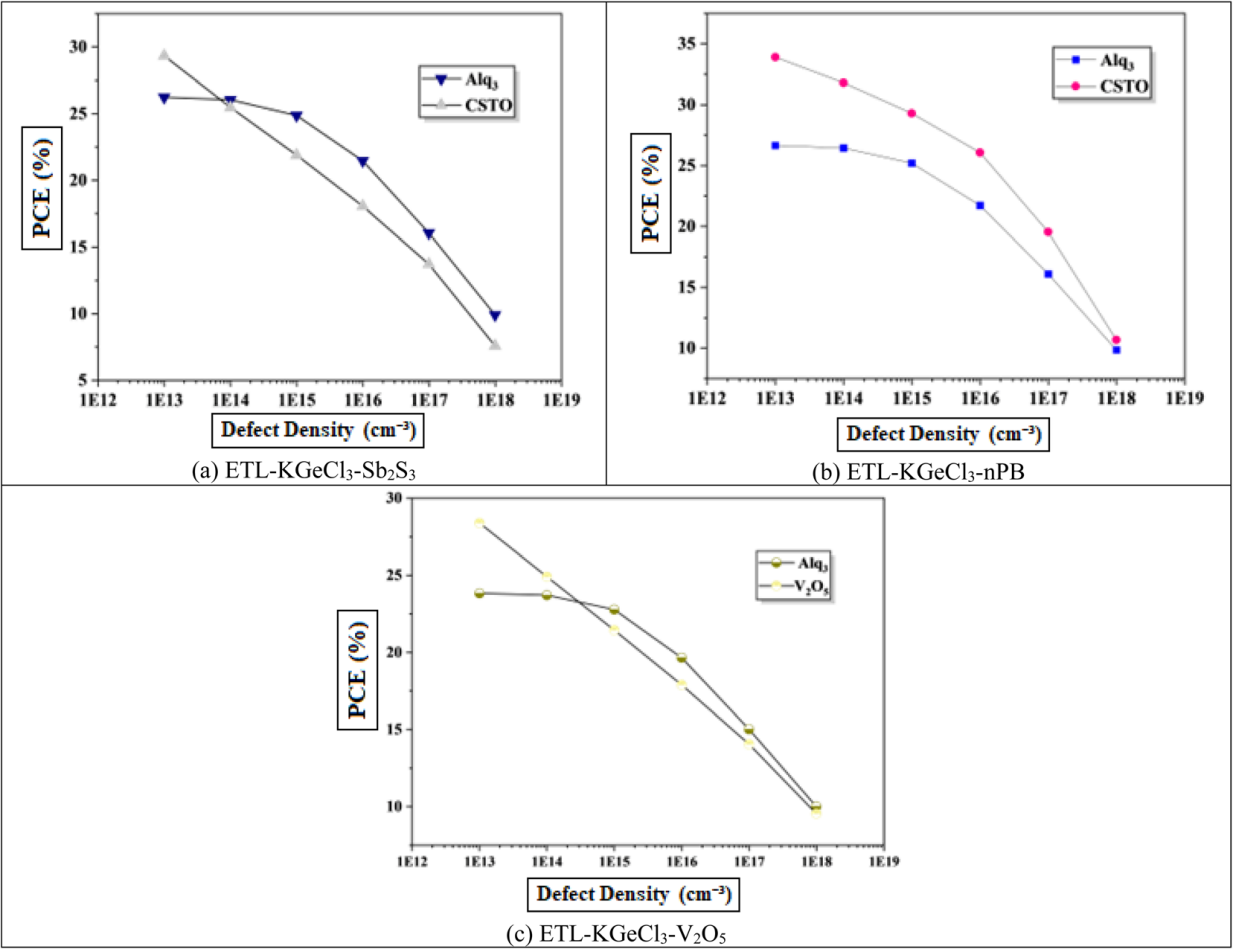
Effect of absorber defects.

Fig. 11 shows the negative effect of increasing defect density in the KGeCl_3_ absorber layer on *V*_oc_ and *J*_sc_. As defect density rises from 10^13^ cm^−3^ to 10^18^ cm^−3^, there is a noticeable decline in PSC performance. This decline occurs because higher defect densities provide more trap states for charge carriers, leading to increased recombination and reduced carrier lifetimes.^45^ In this study, optimal performance across all cell configurations was observed at a lower defect density, highlighting the importance of minimizing defects to maximize PSC effectiveness.

**Fig. 12:**
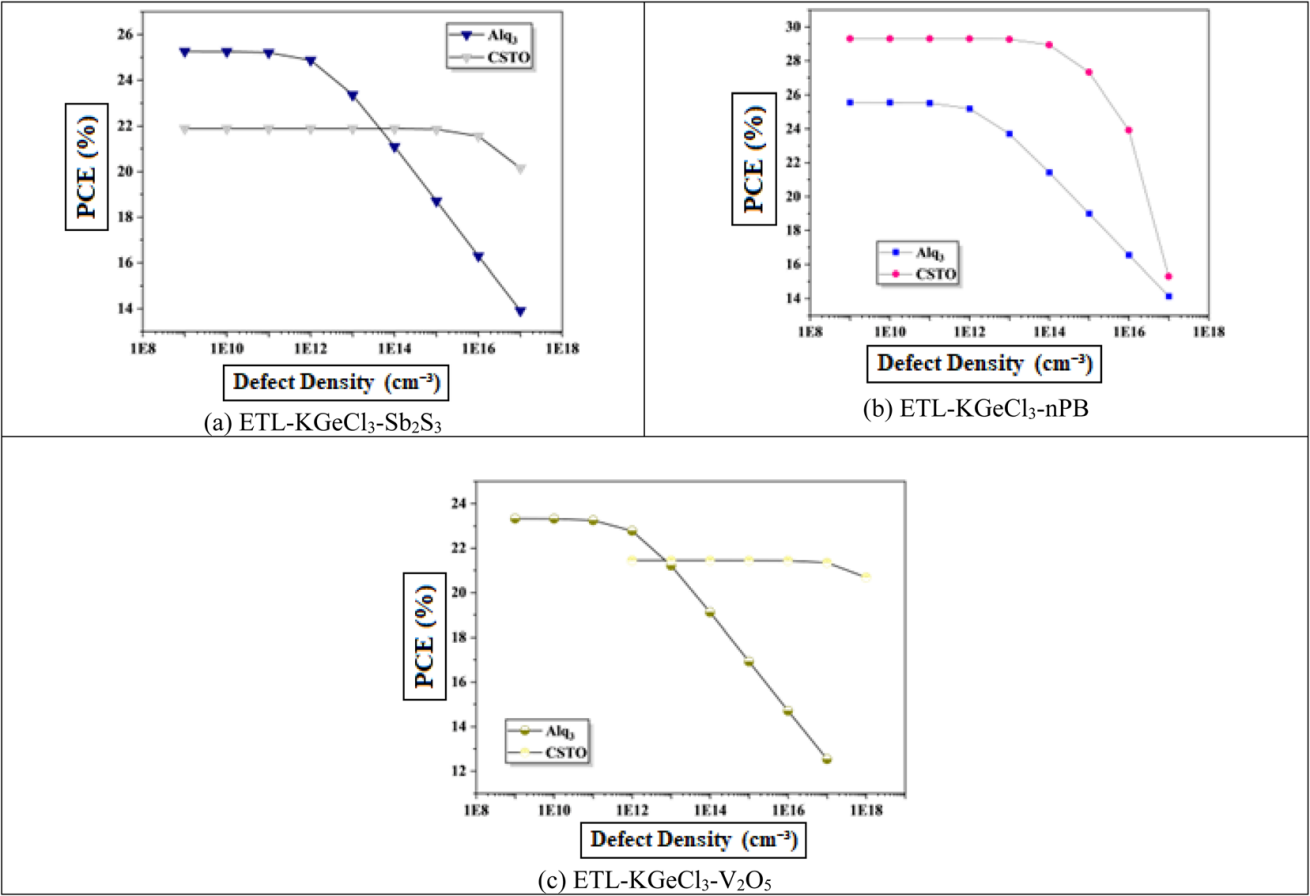
Effect of ETL defects.

Similarly, increasing defect density within the CTLs was also investigated to understand its impact on PSC performance, as shown in Fig. 12. It reveal results similar to that observed with absorber defects. As defect density rises in the CTLs, overall device performance declines. This performance reduction is due to the increased resistance and formation of trap centers within the CTLs, which hinder the movement of photo-generated electron–hole pairs. These additional traps lead to higher recombination rates, thereby reducing charge carrier lifetime and impeding efficient charge transport through the device. Increased defects within the CTLs not only impede carrier mobility but also introduce significant losses in *J*_sc_, *V*_oc_, and PCE.

### 4.4. Work function optimization

The electrodes, as the outermost layers of PSCs, play a crucial role in device performance by providing efficient charge extraction while minimizing energy losses due to resistive heating. For optimal operation, these electrodes must exhibit low resistance and high conductivity to support a high FF and maximize PCE.^47^ Furthermore, electrode compatibility with the adjacent CTLs is essential, as it strongly influences charge extraction efficiency and overall device performance. The work function of an electrode, which represents the energy needed to move a charge carrier from the Fermi level to a reference energy level, is key in determining how easily it can release or capture charge carriers when in contact with the CTL.^48^ The work function difference between the electrode and CTL impacts the effectiveness of charge extraction from the perovskite absorber layer. Proper alignment of the electrode work function with the energy level of the CTL minimizes energy barriers, reducing the chances of charge recombination and enhancing both *V*_oc_ and PCE.

**Fig. 13:**
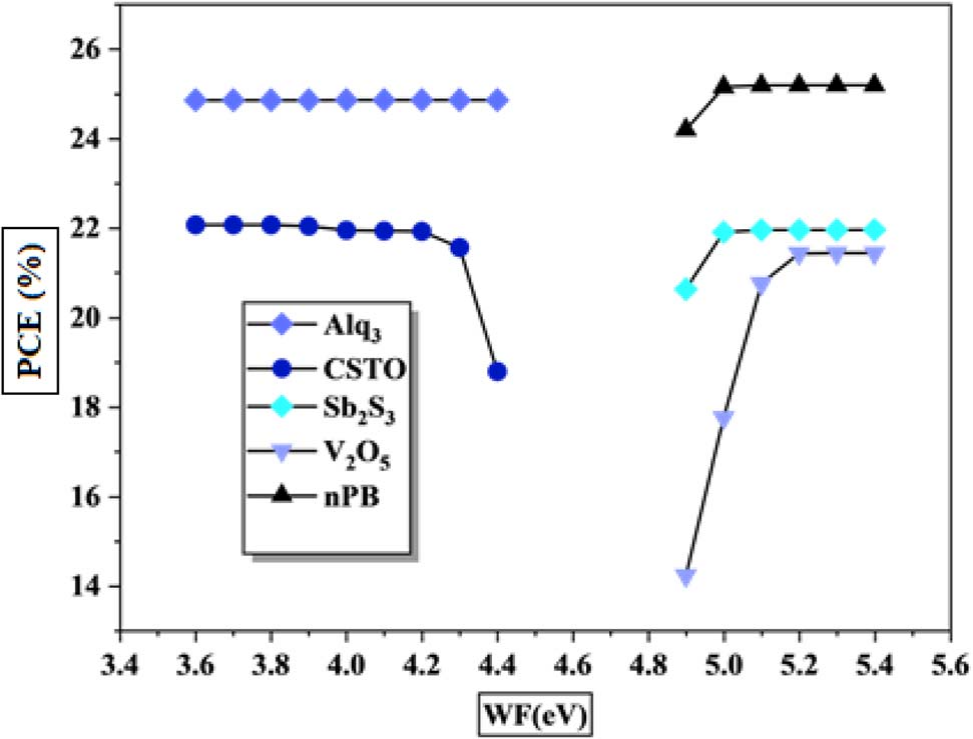
Effect of work function.

In this study, various work function values were evaluated with different CTLs to determine optimal alignment. Fig. 13 shows the effect of varying work functions on CTL performance. Results show contrasting behavior between the ETL and HTL. ETL performance improves as the work function decreases, saturating near 4.4 eV, beyond which further reduction has minimal impact. Conversely, HTL performance improves with increasing work function, reaching saturation around 4.9 eV. This difference arises due to the nature of charge carriers in each layer. The electrons in the ETL occupy the conduction band, which is lower in energy, whereas holes in the HTL occupy the higher-energy valence band. Consequently, ETL materials with a work function below 4.4 eV and HTL materials with a work function above 5 eV demonstrate optimal compatibility for efficient charge extraction in KGeCl_3_-based PSCs.

### 4.5. Reflection layer optimization

In PSCs, a sizable portion of photons, particularly those with longer wavelengths and lower energy, often pass through the active absorber layer without contributing to charge carrier generation.^49^ This spectral mismatch reduces the overall photon utilization efficiency and limits the PCE of the device. To address this issue, introducing a back-end reflective layer (Rb) within the cell structure can be highly effective. The reflective layer redirects these transmitted photons back toward the absorber, increasing their interaction with the active material. This multiple-pass phenomenon enhances the probability of photon absorption in the active layer, resulting in a higher generation rate of electron–hole pairs and improving the overall efficiency of the device.^50^

**Fig. 14:**
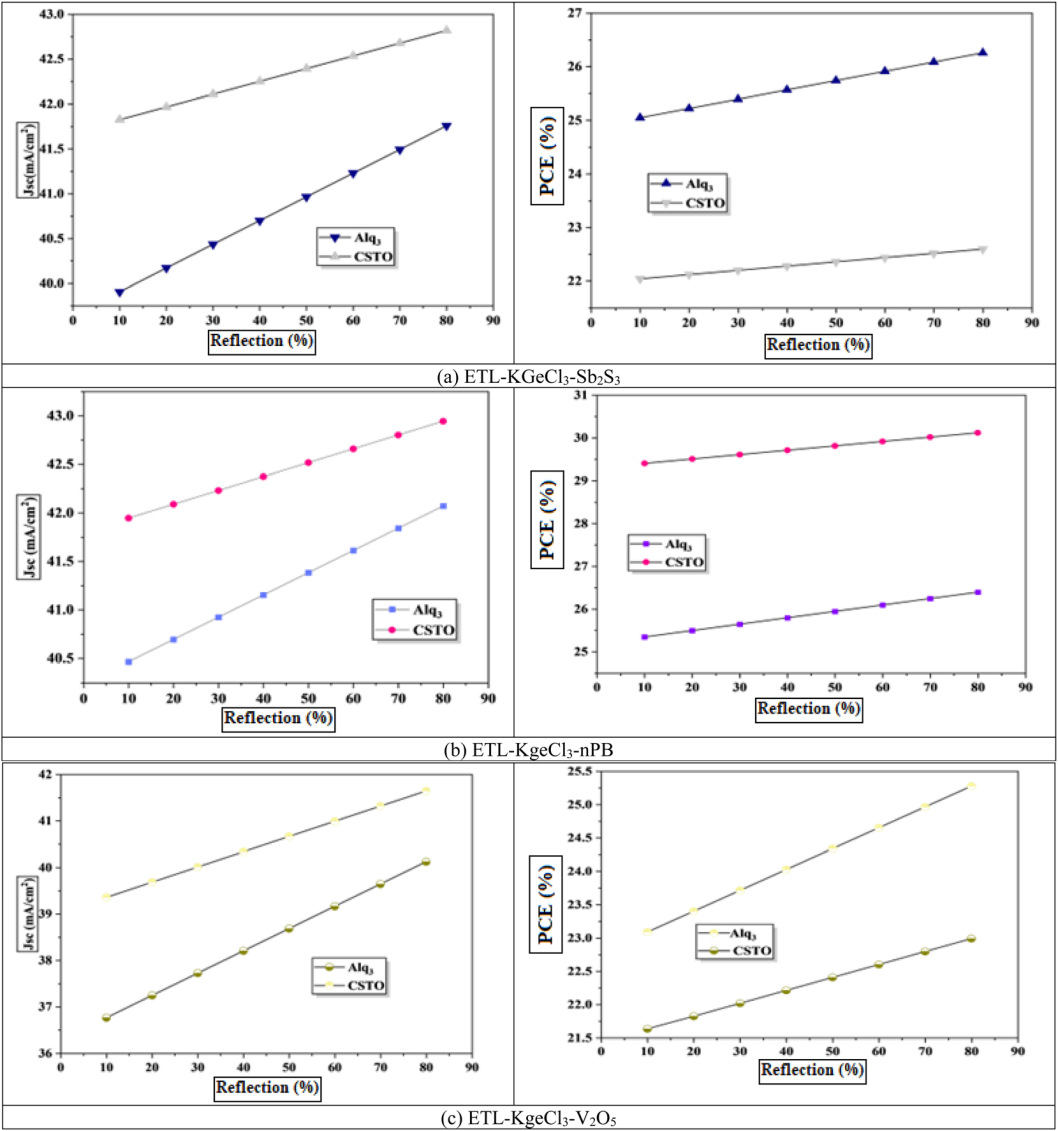
Effect of reflection layer concentration.

To assess the impact of the Rb layer, its reflectivity was varied from 10% to 90%, as shown in Fig. 14. The results demonstrated a clear correlation between increasing reflectivity and improved PCE. At higher reflectivity values, more photons were redirected back into the perovskite layer, boosting light absorption, and increasing the *J*_sc_.^51^ This enhancement in photon utilization highlights the critical role of an optimized back-end reflective layer in improving PSC performance. Additionally, increased reflectivity reduces light leakage from the device, further contributing to efficiency gains.^52^ The observed improvement in PCE with an Rb layer reflects its ability to compensate for the intrinsic limitations of the perovskite absorber thickness, particularly in cells designed with thinner active layers for better charge extraction and reduced recombination losses.^53^ By enabling more effective light management, the Rb layer minimizes the trade-off between optical absorption and electrical performance.

### 4.6. Effect of temperature

**Fig. 15:**
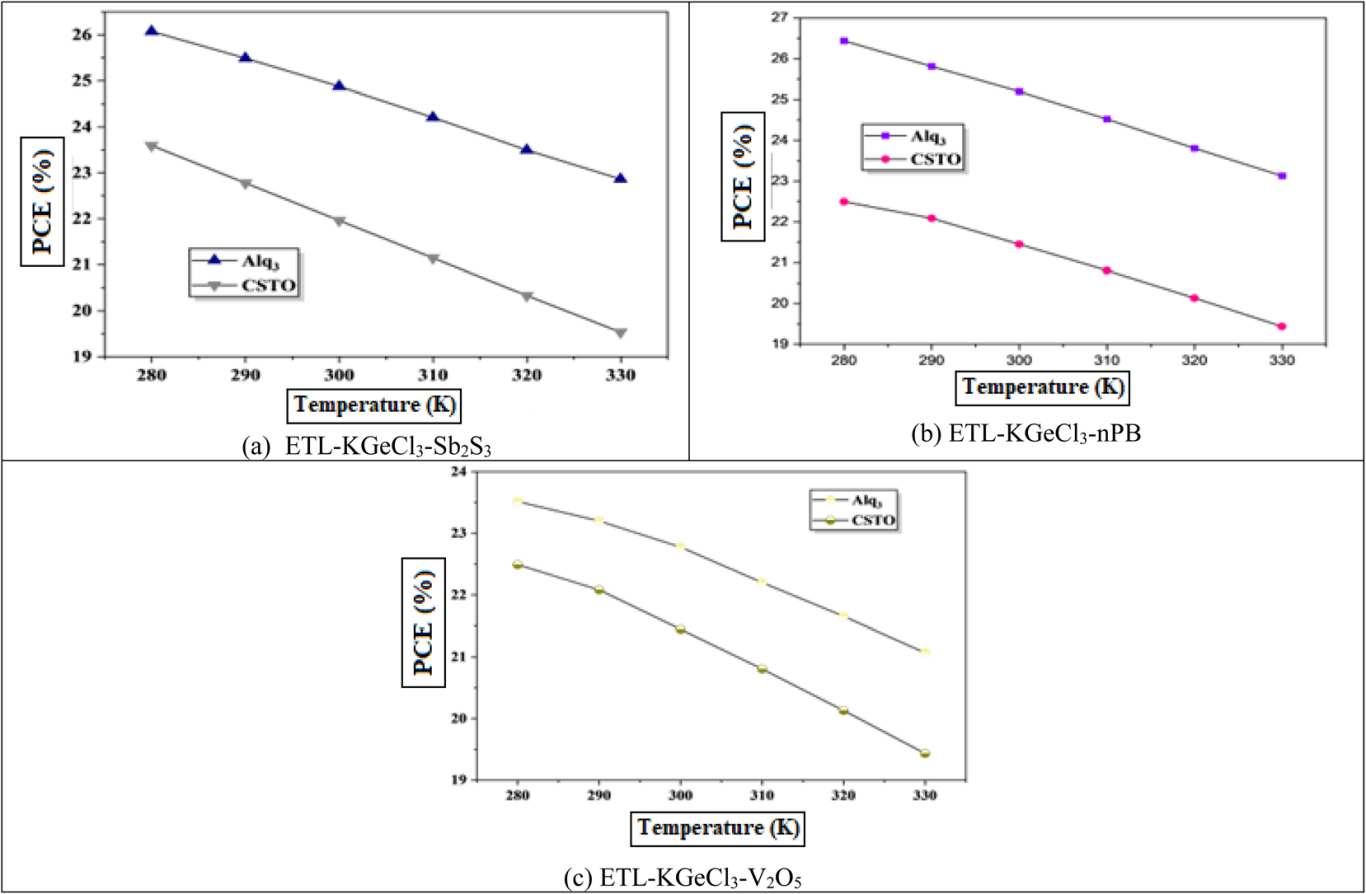
Effect of temperature.

Solar cells typically operate in environments with temperatures exceeding 300 K, which significantly affects their performance. Temperature is a critical factor that directly impacts key device parameters, including *V*_oc_ and PCE.^54^ To investigate the impact of temperature on the efficiency of the KGeCl_3_-based PSCs, the operating temperature was systematically increased, as shown in Fig. 15. The PCE decreases as temperature rises because of decrease in *V*_oc_.^54^ This reduction in *V*_oc_ can be attributed to an increase in reverse saturation current (*I*_o_), as *V*_oc_ and Io are inversely related according to the diode equation:^55^3
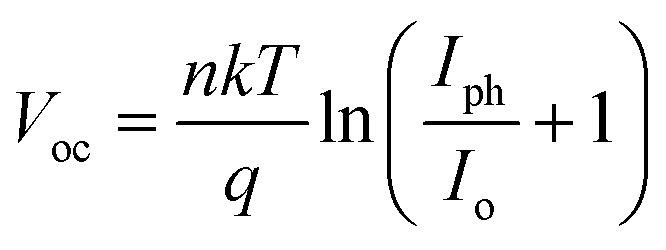
Here, *k* is Boltzmann's constant, *T* is the temperature, *q* is the elementary charge, *n* is the ideality factor, and *I*_ph_ is the photocurrent. As *T* increases, *I*_o_ rises exponentially, reducing *V*_oc_. This effect underscores the thermal sensitivity of perovskite materials and the need for managing temperature to sustain high efficiency.

Higher temperatures also accelerate the recombination rate due to increased series resistance and diminished charge carrier mobility, both of which contribute to reduced conversion efficiency. Additionally, elevated temperatures affect the bandgap energy and conductivity of the perovskite material, further degrading cell performance. These findings underscore the importance of thermal management in PSCs to maintain optimal efficiency and stability under varying environmental conditions.

### 4.7. Optimized results

Table 4 shows the optimized performance and design parameters for the various KGeCl_3_-based PSC configurations. Each structure displays unique optimized values for absorber thickness and doping levels, reflecting the distinct influence of different CTL combinations on band alignment and built-in potential, which in turn affect the device's performance. An optimized thickness of 0.1 μm was maintained for all CTLs, while a doping concentration of 10^20^ cm^−3^ was selected as optimal for the HTLs.

Among the PSCs, the CSTO-KGeCl_3_-*n*PB structure demonstrated the highest PCE at 29.30%, with an absorber thickness of 650 μm and ETL doping of 10^17^ cm^−3^. This result highlights the strong compatibility between CSTO and *n*PB as CTLs with the KGeCl_3_ absorber, achieving superior charge transport and minimal recombination. Other high-performing structures included Alq_3_-KGeCl_3_-*n*PB and Alq_3_-KGeCl_3_-Sb_2_S_3_, which also achieved notable efficiencies, albeit lower than the CSTO-KGeCl_3_-*n*PB configuration.

To contextualize the findings of this study, the performance of the proposed KGeCl_3_-based PSCs is compared with state-of-the-art PSCs reported in the literature. The comparison includes parameters such as open-circuit voltage, short-circuit current density, fill factor, and power conversion efficiency. Table 5 provides a comprehensive comparison of the designed KGeCl_3_-based PSC with other state-of-the-art PSCs. The table is divided into two groups, S. No. 1–6 represent experimental PSCs employing different perovskite materials with high PCE, while S. No. 7–12 focus on KGeCl_3_-based PSCs designed by other researchers and are also discussed in the introduction section of the paper. S. No. 13 is the PSC designed in this study.

**Table 5:** Comparison of state-of-the-art PSCs

S. No.	Structure	*V*_oc_ (V)	*J*_sc_ (mA cm^−2^)	FF (%)	PCE (%)	Ref.
1	FTO/SnO_2_/FAPbI_3_/Spiro-OMeTAD/Au	1.181	25.14	84.8	25.2	56
2	FTO/CdTe-TiO_2_/CsFAMAPbI_3_/Spiro-OMe TAD/Au	1.167	25.61	83.81	25.05	57
3	ITO/SrSnO_3_/FAMAPbI_3_/Spiro-OMeTAD/Au	1.17	25.72	83.67	25.17	58
4	FTO/SnO_2_/FACsPbI_3_/Spiro-OMeTAD/Au	1.2	25.12	83.48	25.24	59
5	ITO/MeO_2_/RbCsMAFAPb(IBr)_3_/LiF/C_60_/BCP/Ag	1.16	26.096	83.82	24.05	60
6	ITO/MPACPA/CsFAMAPb(IBr)_3_/C_60_/BCP/Ag	1.24	24.8	87.5	25.4	61
7	ITO/IGZO/KGeCl_3_/V_2_O_5_/Au	0.67	42.04	75.39	21.23	5
8	FTO/MoO_3_/KGeCl_3_/WS_2_/Au	0.88	41.45	81.76	29.02	6
9	FTO/SnS_2_/KGeCl_3_/Cu_2_O/Au	0. 545	41.91	69.24	15.83	62
10	FTO/SnO_2_/C_60_/KGeCl_3_/Me_4_PACz/Au	1.13	23.47	73.34	19.62	63
11	ITO/CBTS/KGeCl_3_/Ws_2_/Ni	0.679	41.439	78.12	22.01	64
12	FTO/TiO_2_/KGeCl_3_/Cu_2_O/Au	0.90	38.6	82.84	29.03	65
13	FTO/CSTO/KGeCl_3_/*n*PB/Au	0.815	41.804	85.97	29.302	[This work]

From S. No. 1–6, it can be observed that these PSCs utilize a range of perovskite materials, such as FAPbI_3_, CsFAMAPbI_3_, and FACsPbI_3_, paired with optimized charge transport layer combinations. For instance, the cell at S. No. 1, employing FAPbI_3_ with SnO_2_ and Spiro-OMeTAD as CTLs, achieves a PCE of 25.2% due to its excellent *V*_oc_ of 1.181 V and high fill factor of 84.8%. Similarly, the cell at S. No. 2, using CsFAMAPbI_3_ with CdTe-TiO_2_ and Spiro-OMeTAD, achieves a PCE of 25.05% with *J*_sc_ of 25.61 mA cm^−2^ and a FF of 83.81%. Similarly, the PSC at S. No. 3 uses a FAMAPbI_3_ perovskite along with SrSnO_3_ CTL achieving a PCE of 25.17. These examples demonstrate the critical role that CTL selection plays in achieving high efficiency by ensuring proper band alignment, charge extraction, and reduced recombination losses. From these results, it can be concluded that identifying the right CTL combination for KGeCl_3_ is of vital importance to unlocking its full potential, which has been carried out in this study.

The PSCs of S. No. 7–12 represent KGeCl_3_-based PSCs designed by other researchers and an interesting trend emerges. While most cells exhibit nearly identical *J*_sc_ values in the range of 41–42 mA cm^−2^, their *V*_oc_ values vary significantly, resulting in notable differences in FF and PCE. For instance, the cell at S. No. 7, employing IGZO and V_2_O_5_ as CTLs, achieves a *J*_sc_ of 42.04 mA cm^−2^ but has a relatively low *V*_oc_ of 0.67 V, leading to a PCE of 21.23%. Conversely, the cell at S. No. 12, using TiO_2_ and Cu_2_O as CTLs, achieves a higher *V*_oc_ of 0.90 V and a PCE of 29.03%. This variation in *V*_oc_ is directly related to the CTL combinations, as different CTLs result in varying band alignments and band offsets at the heterojunction. These band offsets may produce either a spike or a cliff at the interface, influencing the built-in potential of the cell and ultimately affecting *V*_oc_ and overall performance. For instance, the lower *V*_oc_ observed for cells using IGZO as ETL can be attributed to a less favorable band alignment, which reduces the electric potential across the absorber layer.

From this analysis, it is evident that the heterojunction compatibility between the CTLs and KGeCl_3_ is critical for achieving optimal performance. In this work, emerging and novel CTL combinations were investigated to unlock the full potential of KGeCl_3_. By carefully selecting and optimizing these CTL combinations, the designed PSC in this work, represented by S. No. 13 in Table 5, achieved the highest PCE of 29.302%, surpassing all other KGeCl_3_-based PSCs in the literature. This demonstrates that through proper selection of CTLs and systematic optimization, the performance of KGeCl_3_-based PSCs can be significantly enhanced, paving the way for high-efficiency and environmentally friendly photovoltaic technologies.

## 5. Conclusion

The need for efficient, stable, and environmentally friendly photovoltaic solutions drives the development of KGeCl_3_-based perovskite solar cells (PSCs). This study demonstrated the potential of KGeCl_3_ as an absorber material through a comprehensive optimization process involving various charge transport layers (CTLs). Through systematic variations in layer thickness, doping concentration, defect density, and work function, the study identified the optimal configurations for maximizing PCE and charge transport efficiency. The study found that increased absorber thickness enhances light absorption, improving *J*_sc_, up to an optimal level where recombination begins to counteract these gains. CTL doping optimization further revealed that higher doping in the ETL and HTL increases conductivity, reducing series resistance and boosting *J*_sc_ and *V*_oc_. However, excessive doping levels alter the semiconductor properties, shifting them towards metallic behavior, which hinders charge transport. Additionally, minimizing defect density in both the absorber and CTLs was critical for enhancing carrier lifetime and reducing recombination. Furthermore, the study showed that an appropriate work function is essential for optimal charge extraction, with ETL work functions below 4.4 eV and HTL work functions above 5 eV proving most effective. Introducing a back-end reflective layer with 90% reflectivity improved *J*_sc_ by increasing photon absorption, further contributing to efficiency gains. Among the PSCs, the CSTO-KGeCl_3_-*n*PB structure emerged as the top-performing PSC, achieving a PCE of 29.30%. This high efficiency is attributed to effective band alignment and reduced recombination rates, supported by optimal doping levels that promoted efficient charge separation and transport. Other configurations, such as Alq_3_-KGeCl_3_-*n*PB and Alq_3_-KGeCl_3_-Sb_2_S_3_, also demonstrated high PCEs of 25.19% and 24.87%, respectively, displaying the influence of CTL selection on device performance. These findings not only validate the potential of KGeCl_3_ as a viable absorber material but also provide insights into design strategies for high-efficiency PSCs, contributing to the development of sustainable and clean energy technologies.

## Declaration

During the preparation of this work the authors used Grammarly tool in order to improve English language and proofread. After using this tool/service, the authors reviewed and edited the content as needed and takes full responsibility for the content of the publication.

## Data availability

Data will be provided upon reasonable request from authors.

## Conflicts of interest

The authors declare that they have no competing interest with any party.
